# Death doula working practices and models of care: the views of death doula training organisations

**DOI:** 10.1186/s12904-023-01200-w

**Published:** 2023-06-23

**Authors:** Deb Rawlings, Lauren Miller-Lewis, Jennifer Tieman, Kate Swetenham

**Affiliations:** 1 Centre for Palliative Care Death and Dying, College of Nursing and Health Sciences, Flinders University, GPO Box 2100, Adelaide, South Australia 5001 Australia; 2 College of Psychology, School of Health, Medical and Applied Sciences, CQ University Australia, Adelaide campus, South Australia Australia; 3 Health Programs and Funding Branch, Department for Health and Wellbeing, Adelaide, South Australia Australia

**Keywords:** Death doula, Palliative Care, End of life, Models of care

## Abstract

**Background:**

The role of death doula has emerged in recent years, arguably as a result of overwhelming demands on carers, healthcare professionals and service providers in end-of-life care. Death doulas work independently without governing oversight and enact the role in various ways. The main driver of this evolving role is the organisations that train them. The aim of this study was to examine death doula training organisations’ views with regard to DD business models, incorporating the death doula role into other existing models of care, and role enactment.

**Methods:**

An electronic survey was administered to 15 death doula training organisations in 5 countries asking additionally that they disseminate the survey. Responses were received from 13 organisations, based in Australia (n = 4), the US (n = 4), Canada (n = 2), the UK (n = 1), Sweden (n = 1) and New Zealand (n = 1). This paper provides the qualitative findings from four open-text questions posed within the survey related to models of care.

**Results:**

Qualitative data analysis was inductive, themes were determined in relation to: (1) standardised business model for death doulas, (2) death doulas incorporated into existing models of care or existing funding options, (3) death doulas who volunteer their services rather than charge money, and (4) role specialisation such as has occurred with birth doulas.

**Conclusions:**

The death doula role has the potential to be formally recognised in the future under national registration schemes, accompanied by death doula training required via certification. Until such time the death doula role will continue to evolve much as the birth doula role has, organically and unstructured. How and if death doulas are incorporated into existing models of health or social care remains to be seen as the organisations that train them push for independence, flexibility and fiscal independence.

## Introduction

The last months or years of life are often spent at home interspersed with visits or appointments to hospital, the Emergency Department and to a General Practitioner [1]. Family, friends, and others taking on caring roles join health care providers in meeting physical, psychosocial, emotional and spiritual care needs. Those nearing death can have complex care needs met via a mix of health and social care services including volunteers, community pharmacists, local councils and churches [1].

Death doulas (DDs; also known as end-of-life doulas), described as non-medical advocates or support persons for those at the end of life, are entering the end-of-life space and taking up various roles in the employ of families [2]. As birth doulas (BDs) work with women in pre- and post-natal care, so DDs work with clients and / or families at the end of life. The doula role generally has unofficially existed for many years, initially in the form of a local woman who helped with birthing babies and laying out dead bodies in the local community [3].

The DD role remains unregistered globally (as does the BD role), with no standardised or mandated training, although DDs will usually attend one or more courses. The variability in how they enact their role makes it difficult to estimate the cost (if any), how often they will attend the client and family (at their discretion), or services provided. The latter may encompass providing companionship, helping with advance care planning, providing spiritual support, providing respite for family, providing physical care, maintaining deathbed vigils or even conducting the funeral [4]. The DD role is enacted in various ways, presenting an interesting conundrum with the growing numbers of DDs being trained, as seemingly not all DDs can or want to provide all aspects of death care or work. For example, some charge a fee for service and others volunteer, some work exclusively with the client, others exclusively with the family, and some with both [4]. This diversity, while seen as a strength by some advocates in that it provides an individualised flexible service [5], also means that a health care professional (HCP) working alongside a DD, or a consumer seeking a DD service cannot necessarily expect the same service delivered by any two DDs.

The word ‘doula’ has been used more formally by BDs, appearing in the academic literature since the 1970’s. It is only in the last 5 or 6 years that the role of DD has started to be formally researched in the form of an early systematic literature review [2], and a more recent scoping review [6], highlighting that published evidence about the role and impact of the role is limited, suggesting the need for more rigorous studies [6]. Research has also been undertaken with DDs themselves via an initial survey and interviews conducted by the authors [4, 7]. Findings from the survey confirmed there is diversity in training, experience, skills and role enactment of DDs. One point of consensus from the survey is that DDs want the role to be taken seriously [4]. There was ambiguity among DDs about whether the DD role should be formally registered in the same way nursing roles are and/or connected to a minimum standard of training and education [4]. In interviews with DDs, it was noted that there was some overlap between the DD role and that of some HCPs [7].

Other authors in conducting interviews with DDs also describe the concept of role evolution (a “community-entrepreneurial social movement”) and view the diversity of DD role enactment not as a limitation but as a self-reflexive and foundational component of practice [8]. An exploratory study was also undertaken to document DD demographics, backgrounds and service provision noting again that it is a new and little understood form of care with challenges to gaining recognition and acceptance [9]. A compassionate communities DD model has been developed whereby a DD is “engaged as part of the overall end-of-life support network” [10], and research has been undertaken relative to a DD as a personal service job striving for occupational legitimacy [11]. The authors have also started to consider the views of DD training organisations [5].

Prior research suggests that DD training organisations are the primary driver shaping the role [4, 7, 12]. It is yet unknown how the role will continue to evolve as is the possibility of incorporation into mainstream healthcare. Given the gap in knowledge regarding the role and views of DD training organisations, we recently conducted the first international study of DD training organisations and asked participants questions regarding the DD role [5]. These training organisations are not only where DDs receive their education, but also their work philosophy and approach, and in addition, may also be a source of ongoing support and advocacy for those they have trained. We found that while the doula organisations were run similarly, and there were several topics consistent across their curricula, there were points of difference regarding the qualifications of trainers, and some organisations strongly valued the unique aspects of their curriculum content. Trainers’ views were mixed about the way to proceed with registration of the Death Doula role.

The existing literature describes a role that is in its early foundational stages, and one that is continuing to evolve. DDs for the most part work independently without governing oversight and many are keen for this to continue. If DDs are professionalised (via mandated registration, education) in order to instil confidence and credibility within the healthcare sector, then this will undoubtedly be led via the organisations that train them. The voice of the families who have engaged a DD remains silent within the literature, and a study is underway by the authors to address this gap.

The aim of this current study was to examine DD training organisations’ views about DD working practices and models of care. Specifically, we sought to answer the following question: What are the views and perspectives of DD training organisations about DD business models, incorporating the DD role into other existing models of care, role enactment (specialisation of the role), and the DD as a volunteer?

## Methods

### Ethics approval

Ethical approval was obtained from the Flinders University Research Ethics Committee (Project: SBREC7933) to conduct a DD organisational survey internationally. The survey invitation was administered electronically via publicly available email addresses and included a participant information sheet, where it was made clear that consent was provided by completing the online form.

### Participants and procedure

Four open-ended questions related to the working practices and roles of DDs were posed to DD training organisations in an online survey administered from 4th August to 29th August 2021. These questions (each shown in the results section below) were part of a larger study reported elsewhere [5]. Fifteen DD training organisations in 5 countries: Australia (AUS), New Zealand (NZ), Canada (CAN), the United States (US) and the United Kingdom (UK) were approached. They were asked to further disseminate the survey to other DD training organisations via a snowball sampling recruitment approach [13]. Organisations were considered eligible to participate if they provide DD training.

Responses were received from 13 organisations, based in AUS (*n* = 4), the US (*n* = 4), CAN (*n* = 2), the UK (*n* = 1), NZ (*n* = 1) and Sweden (SWED) (*n* = 1). From the 15 organisations originally identified and contacted *n* = 6 (40%) responded, with the additional *n* = 7 organisations sourced via the snowball sampling approach [5]. The authors have assumed that the manager or owner of the DD training organisation completed the survey, but this cannot be verified [5].

### Qualitative analysis

The authors have researched extensively on the DD role, and in order to avoid introducing preconceived bias in analysis [14], an independent research assistant (not a DD) performed qualitative data analysis on responses to these four open-ended questions from the larger survey [5]. Data was analysed using an inductive approach [15], with participant responses driving the analysis. This involved reading through all responses and deriving themes via open coding and subsequently axial coding [16]. Two of the researchers (authors DR, LML) further refined the themes and sub-themes via cross-verification with an iterative approach. This subtheme refinement was undertaken by two of the authors who have training in palliative care, public health and psychology but are not DDs. As described by Graneheim et al. [17], this approach to qualitative content analysis is more descriptive rather than thematic, in that less interpretations were made from the participant responses and a broader thematic analysis was not completed.

Exemplar quotes are provided for each theme with excerpts taken and potentially coded across more than one theme.

## Results

The thirteen organisations are anonymous, but some details are provided in Table 1. While the survey was relative to death doulas, it is feasible that the numbers of birth doulas trained was also included in the number provided by some organisations who provide training to both (i.e., the numbers provided are higher than expected over the length of time training).

**Table 1:** Organisation details

Organisation	Country	Years providing training	Number of DDs trained over the past year
1	Australia	1	12
2	Australia	20	150
3	The US	5	400
4	Sweden	< 1	0
5	New Zealand	2	10
6	Canada	5	> 1000
7	Canada	3	50
8	The UK	2	24
9	The US	1	50
10	Australia	0	0
11	The US	16	200–300
12	The US	1	5
13	Australia	5	276

Further information on the DD training organisations who responded to these questions can be found in the previous study undertaken by the authors: An international survey of Death Doula training organisations: The views of those driving Death Doula training and role enactment [5].

The responses to these four open-text questions provide differing perspectives on the nature of the business of being a DD, and of providing services which could affect how able and prepared DDs are to establish their role in the sector. This diversity in views was consistent across operational aspects such as standardisation of business models, role enactment (volunteering and specialisation) and potential funding and reimbursement frameworks. This non-consensus aligns with responses to questions posed to the same organisations regarding other aspects of the role and the training offered [5], and to the DDs themselves when questioned [2, 7].

### Question

What are your thoughts on a standardised business model for doulas?

Responses to this open-ended question were categorised into 3 themes: (1) rejected business standardisation, (2) supported business standardisation and (3) did not support this idea outright, not sure or expressed concerns. (Table 2).

**Table 2:** Business models for Death Doulas

Theme	Respondent	Response
**Rejected business standardisation**	Organisation 2 (AUS)	*“It will get sucked back up by the system”*
	Organisation 3 (the US)	*“No. Each doula is as unique as the family served”*
	Organisation 10 (AUS)	*“NO thanks* “
	Organisation 11 (the US)	*“not going to happen. doulas are too independent and they have too many varied skill sets. this is not a typical health care role. I have wondered about this since I started seeing more and more people come into this since 2010 and I still dont see it happening”*
	Organisation 12 (the US)	*“No, owning a business is not part of being a doula. It can, but its not the focus. Businesses should employ doulas, not certify them and make them the customers”*
**Supported business standardisation**	Organisation 6 (CAN)	*“I agree* “
	Organisation 13 (AUS)	[…] “That being said, a standardised business model across Australia would provide transparency for clients. Having doula services entrenched as main stream and funded accordingly through the NDIS*, aged care packages and home and community care funding or itemised through medicare** / health funds etc would be beneficial to all and have this service accessible to people of all socio-economic backgrounds. The emphasis should be on the conduct and practices of the doulas rather than their business model or structuring. We envisage and anticipate that doulas would agree to abide by a standard Code of Conduct associated with holding a current registration to provide end of life doulas services with the professional body. That would be linked to holding requisite insurance, continuing education requirements, duties, ethics, responsibilities, conflict resolution, adherance to required legislations like privacy/confidentiality etc.”
**Did not support outright, not sure or expressed concerns**	Org 1 (AUS)	“Standards of practice and ethos, yes (similar to registered art therapists with ANZACATA***, for example)”
	Organisation 4 (SWED)	*“Some of it perhaps yes but still, every country regardless if they happen to have for example English as a common language will still have differences in culture, laws etc.* “
	Organisation 5 (NZ)	*“I think this will occur naturally over time. The word standardised just crushes the joy and magic out of things”*
	Organisation 7 (CAN)	*“Mixed. not sure how this would look but standardised rates would add clarity and validation”*
	Organisation 8 (the UK)	*“Not sure”*
	Organisation 9 (the US)	*“If it goes that route, then that is fine– there will be another grassroots movement to take it’s place”*

To note: *NDIS is the Australian National Disability Insurance Scheme; **Medicare is a national insurance scheme that provides free or subsidised healthcare for all Australians; ***ANZACATA is the peak professional association for creative arts therapies in Australia, New Zealand and Asia

Five organisations rejected business standardisation outright while two openly supported it. The other six organisations either did not support this idea outright, were unsure or expressed concerns.

**Question**: We have considered that doulas could be incorporated into existing models of care or existing funding options. What are your thoughts on this? (For example: to incorporate doulas into a palliative care model in the same way that volunteers in hospice or hospital are managed. Doulas to become an option at a health care staffing agency. Doulas to be added as a partially funded option under private health insurance).

Responses to this open-ended question were categorised into 5 themes: (1) supported incorporating DDs into models, (2) supported incorporating DDs into models but with caveats, (3) DDs are not the same as volunteers, (4) DDs to remain independent and (5) DD role to remain non-medical (Table 3).

**Table 3:** Death Doulas incorporated into existing models of care

Theme	Respondent	Response
**Support incorporating DD into models**	Organisation 2 (AUS)	*“I think this could be good”*
	Organisation 4 (SWED)	*“Yes, a great complment* (sic)“
	Organisation 5 (NZ)	*“Yes to all that!!!“*
	Organisation 6 (CAN)	*“I agree this is the right direction”*
	Organisation 12 (the US)	*“Yes, we are working on becoming a licensed home health agency exclusively for EOL doulas. Not hospice.”*
**Support but with caveats**	Organisation 7 (CAN)	*“I would love to see all of the above but with the doulas at the table to help determine the scope’s boundaries”*
	Organisation 8 (the UK)	*“Yes a hood (sic) way forward as long as the purpose of doulas os (sic) not lost”*
	Organisation 11 (the US)	*“I love all this but you have to be careful how you do it. Its too much to go into here. It is an excellent idea but you have to decide what the main use you want them for in each setting. thats very important. it may be another role will provide what you want”*
**DDs are not the same as Volunteers**	Organisation 1 (AUS)	[…] “I don’t like the idea of putting doulas into a category that contains (or is analogous to) volunteerism […] Funding under private health care yes, however Medicare* will not take on more categories of work”
	Organisation 9 (the US)	*“Sure… so, a glorified Hospice volunteer? It gets tricky… I’m afraid there would be less Hospice volunteers if there is a demand for a paid position as a death doula. It might harm the Hospice volunteer model… It’s all a great unfolding mystery isn’t it?* “
	Organisation 13 (AUS)	*“There is much discussion to be had about the ‘same way that volunteers in hospice/hospital are managed’. The doula role needs to be viewed, and is actually, a specialist role and not simply as an added skill to incorporate into the role of a volunteer / paid carer” […]*
**DD to remain independent**	Organisation 3 (the US)	*“It’s a slippery slope of government regulation and oversight of the profession. Should never be mandated that doulas participate if they want to serve clients who can do private pay […] But turning us into another cog in the machine undermines the very essence of the role. I get the desire to serve lower income families who might not be able to do private pay but this can be addressed through nonprofit options rather than government pay” […]*
	Organisation 10 (AUS)	*“I think that Doulas need to remain independent of any system and be hired by the dying person and/or their family. I would love to see private health insurance companies see the value in what we do and offer rebates for their customers” […].*
	Organisation 13 (AUS)	[…] *“Cautious about doulas being ‘hired’ by hospitals and other institutions etc as they will have to adhere to policies and procedures of the employer which may compromise their capacity to advocate for the values and wishes of the individual client and those close to them” […].*
**DD role to remain non-Medical**	Organisation 1 (AUS)	*[…] “I’m also not convinced that situating end of life doulas into a medical model of practice is beneficial” […]*
	Organisation 12 (the US)	*“Hospice works under physicians orders, doulas should not”*
	Organisation 13 (AUS)	*“Doulas already deliver palliative care (except the medical aspect)”*

*Medicare is a national insurance scheme that provides free or subsidised healthcare for all Australians

Interestingly, eight organisations supported incorporating DDs into existing models (although 3 with caveats). This appears surprising given this could lead to DD practices potentially becoming more standardised, a concept lacking support in the responses to the previous question. Three organisations did not see doulas as acting at the same level as volunteers (i.e., DD ‘*is actually, a specialist role*’). Three organisations would rather keep the doula profession independent or private with no government regulation. Three organisations also expressed a preference for the doula role to remain non-medical.

These responses again highlighted areas of friction and uncertainties that could arise if DDs were connected to existing models. Some saw inclusion impinging on freedoms to practise independently as a DD. One organisation (organisation 12) while noting that they are working to become a DD specific home healthcare agency, stated that DDs should not be under the management of doctors. Another (organisation 13) stated that DDs being hired by institutions implies adherence to policies and procedures which may compromise the ethos of advocating for clients. Two other organisations (1 & 10) felt that a health care staffing or medical model would see clients unable to choose their own DD and subsequently lose continuity of care, perhaps seeing the non-medical DD role subsumed and ultimately lost.

### Question

What are your thoughts on the doulas who volunteer their services rather than charge money?

Responses to this open-ended question were categorised into 3 themes: (1) it devalues or undermines the role (DD are worthy of payment), (2) support for DD as a volunteer and (3) individual choice (Table 4).

**Table 4:** Views of Death Doulas who volunteer

Theme	Respondent	Response
**Devalues or undermines the DD role, worthy of payment**	Organisation 1 (AUS)	*“I think this devalues the field in general - and is dangerous at many levels forpractice […]. Volunteers are often perceived as dispensable, unimportant, valueless […] and makes it harder for all of us to maintain a professional identity […]. Have a service fee set, include a sliding scale if you like, but do not set yourself up to be worthless”*
	Organisation 6 (CAN)	*“I think it undermines their training and effort that it takes to do this job”*
	Organisation 7 (CAN)	*“This is a place of privilege. I have a knee jerk reaction to any compassionate, gendered care being designated to the volunteer sector”*
	Organisation 10 (AUS)	*“I think it’s fair to do both. But i do believe that we need to value this incredible service”*
	Organisation 12 (the US)	*“They are being exploited and hurting the profession from being taken seriously in the medical community”*
	Organisation 13 (AUS)	*“It is a profession, a role, worthy of reimbursement and most people need to earn a living. This is a fee for service role and whilst doulas may not be ‘experts’ and ‘consultants’ they are providing substantial emotional and often spiritual labour. There will always be a need for pro-bono work, to support disadvantaged folk AND there are ways that can be done without doing all volunteer work”*
**Support DD as volunteer**	Organisation 3 (the US)	*“Perfectly fine. Again, I am for freedom of practice and there is room for all of us. The idea that someone who volunteers their services is undermining the value of the role is rooted in a scarcity mindset” […]*
	Organisation 5 (NZ)	*“If they can it is lovely that they can”*
	Organisation 9 (the US)	*“Yes)“*
	Organisation 11 (the US)	*“there is a need for doulas in every level of business, volunteer org and community and family. there needs to be doulas everywhere from every angle. they are all great”*
**Individual choice**	Organisation 2 (AUS)	*“If that is sustainable for them, thats fine, but it needs to be dependant on each persons situation”*
	Organisation 3 (the US)	*“I teach doulas to value themselves and their services and then learn to make THAT case to prospective clients. If someone else wants to volunteer, that’s up to them”*
	Organisation 4 (SWED)	*“Up to each and everyone but even to charge just a dollar and draw up a contract, makes it more professional and better for both parties if for example one wants to get out” […]*
	Organisation 8 (the UK)	*“People choice”*

The results show that 6 organisations expressed that DDs who volunteer can lead to a devaluing of the DD role as a ‘trained’ profession, and the DD role is one worthy of payment and should be valued as a service. Four organisations expressed support for DDs who volunteer and 4 organisations also stated that the choice to charge for service or not should be up to the individual DD.

### Question

What do you think of specialisation such as has occurred in midwifery (e.g., stillbirth or abortion doula) and now in dementia (dementia doula)?

Responses to this open-ended question were categorised into 3 themes: (1) agree with DD role specialisation, (2) disagree with DD role specialisation and (3) unsure about DD role specialisation (Table 5).

**Table 5:** Views on Death Doula specialisation

Theme	Respondent	Response
**Agree with DD role specialisation**	Organisation 1 (AUS)	*“Love it. Quite appropriate for someone who is looking for support from a person who genuinely understands their lived experience/s”*
	Organisation 2 (AUS)	*“good idea”*
	Organisation 5 (NZ)	*“It’s a good thing”*
	Organisation 6 (CAN)	*“I agree that there are many areas a doula can work in”*
	Organisation 7 (CAN)	*“I think it’s fantastic. It helps streamline the awareness of the multi-faceted role of the doula”*
	Organisation 8 (the UK)	*“Specialists shoild (sic) be welcomed if they have extra learning skills and qualification to support it”*
	Organisation 9 (the US)	*“Sure, not every one is tailored to every aspect of deathcare”*
	Organisation 10 (AUS)	*“We are already offering abortion doula training and stillbirth is covered in our foundation training. We also have several other Masterclasses in development”*
	Organisation 11 (the US)	*“i love doulas differentiating themselves so they can really hone in on that specialty”*
	Organisation 13 (AUS)	“I think it makes perfect sense…whilst a doula is a doula and it’s a support role - there are specific skills, education, practices, laws to each arena and laws that govern each area of practice. There are many potential specialist roles for doulas. MS*, MND**, terminally ill children etc, where some additional training and expertise would be useful. Particularly counselling and family support skills”
**Disagree with DD role specialisation**	Organisation 3 (the US)	*“I think it’s kind of silly to have labels for these specialties. Doulas can just list the support services they offer without having to invent a new name for it. But whatever…”*
	Organisation 12 (the US)	*“Its a marketing gemick (sic). A doula should be able to support the clients, not diagnosises* (sic)”
**Unsure about DD role specialisation**	Organisation 4 (SWED)	*“Both yes and no - we should all have a little knowledge but as a doula you’re also a organizer and as such, you can always bring in specalists* (sic)”

*MS – Multiple Sclerosis **MND – Motor Neurone Disease

Most organisations (*n* = 10) supported this idea, with some expressing that this helps doulas highlight their specialist areas of care. Two organisations disagreed, saying that these specialist labels were not useful, that doulas should be there to support clients, not the diagnosis and one was unsure.

## Discussion

Previous research into the DD role has demonstrated that this is a continually evolving role which is not well understood, in part due to the diversity in what is offered. As an emerging role, the DD training organisations are particularly influential while the status of this workforce is being considered and with limited published evidence of its’ impact on patients, families, and health and social systems [2, 4–11]. This study captures the unique views of DD training organisations and as such adds to the emerging body of literature as an important step in understanding the DD role. The findings of this study regarding working practices and models of care as articulated by DD training organisations highlight some considerations that may need to be addressed as part of the evolution of the role. This also reinforces the dichotomy of views between the organisations themselves in how to take their sector forward, and whether standardisation or professionalisation of the role is warranted or wanted.

Many DD organisations articulate the value of individuality in the role and how it is enacted, viewing standardising the business model with rules and regulations as negating a DD’s ability to be flexible and responsive. For some, this may be restrictive or authoritarian and not in the ethos of the DD philosophy or approach. Standardisation may be seen as a restriction on a DDs ability to respond to client’s individual needs which is best protected by minimising requirements or regulation. It may also be a reflection on the preferences and skills of individual DDs enabling them more autonomy in choice of service provision. A lack of standardisation could result in a lack of consistency and clarity regarding client care with consumers not able to expect the same service from two different DDs or uncertain about how to integrate services to ensure they have all their needs met. From a healthcare service perspective, organisations may have difficulty in informing patients or the community about DD services in the absence of a common description of what it is that DDs do. Clarity in role definition would need to be considered going forward if the role is to be broadly promoted and if consumers and clients are to be able to make an informed choice when engaging someone to come into their home.

The interface between DD work and existing models of care provision is of significant interest. So how do DDs encourage improved collaboration and increasing recognition for the value of their work while preserving their freedom of practice? According to Flaherty and Meurer [18], recognition of DDs as a legitimate health care profession is an important initial step, so arguably movement into freedom of practice would then be a natural progression. However, the concept of professional practice in relation to DDs has in itself been challenged [12]. While many DDs use the words ‘profession’ and ‘professional practice’ in referring to their role, some nursing organisations in Australia delineate between professional roles or practice (nursing) and the non-medical (DD) role, arguing that the use of the term ‘scope of practice’ is clinical or medical [12].

Seemingly then, models of care from the perspective of some of the DD training organisations would need to affirm how DDs see their role within the health and social care systems and not just be incorporated, without forethought, into current systems. It is likely that working as an independent contractor via private health insurance or a model where DDs can charge their own fees rather than have them imposed by an organisation may be more acceptable.

Lessons lie in initiatives developed over many years where BDs have been working alongside midwives [19]. Of interest and relevance here is that while Mottl- Santiago and colleagues describe the BD model as a standard maternity intervention [19], they also say that BDs are not routinely used, partly due to the lack of knowledge regarding the role, the lack of standardised training or certification and no reimbursement mechanisms. This reflects our findings in the DD space with a clear need for ongoing work. These authors also state that some American states reimburse BDs via Medicaid (a public health insurance program for people with low income) although there are barriers to this process with recommendations for alternative funding mechanisms [20]. Strauss and colleagues [21] had earlier recognised the potential of BD funding via private insurance, Medicaid, and funding of public programs at city, state and federal levels while Gomez and colleagues [22] consider BDs as contractors and as employees (on an hourly basis with benefits such as sick leave) with the latter a more acceptable alternative by the BDs themselves. The potential to incorporate DDs in a similar way into current or not yet established models of end-of-life care cannot be discounted without an idea of what that might look like in practice.

Similarly, the specialist palliative care sector recognises integration of specialist services with community care [23], as does the more recent emergence of the compassionate communities movement [24]. Examples of specific contextual DD models lie in the palliative care doula model for expert palliative care nurses in a volunteer ministry role [25], and the Mallon model [10], a standardised compassionate community micro-model for caring for someone in the domestic environment. Other similar roles incorporated into health systems or supported by them include patient navigators [26] and Community Health Workers [27].

An important consideration when promoting the integration of new roles into existing systems can again be seen in midwifery, where not all midwives accept the BD role while others embrace it [22–30]. We found that DDs are currently regarded by HCPs in Australia in this way, with challenges regarding acceptance of the role, working models of care (collaborative or not), and role enactment [3]. A key recommendation to collaborative working relationships going forward is ongoing communication [29], and in Australia a DD roundtable was held in 2021 to bring together key stakeholders for a preliminary discussion and ongoing collaboration [12]. Similarly, Neel and colleagues [30] speak of the need for mutual respect, education about the BD role and role clarification or definition between doulas and midwives.

Hybrid fiscal or payment arrangements have been previously noted by the authors that include volunteering [3]. Also noted previously is the perceived overlap of the DD role with that of palliative care volunteer, [31] but it was important in this study to elicit the views of DD training organisations regarding DDs who volunteer their services. Again, similar to the DDs themselves there was no consensus on the DD volunteer with some considering that it devalued the DD as a ‘trained’ profession which is worthy of payment while 4 organisations expressed that the choice to charge for service or not should be up to the individual DD. This view, while speaking again to autonomy and choice in role enactment, also does not allow for consistency in role enactment.

Working under the auspices of an organisation would allow consumers equity of access to DDs, as their pay would be via the organisation (e.g., as with palliative care volunteers), but this in itself poses restrictions in role enactment and threatens the independence that DDs wish to maintain. McLeish et al. [29], describe a community volunteer BD program with formally trained and supervised doulas, and Low et al. [32], describe a similar model whereby trained BDs work in the community, matched with pregnant women by the program. It is difficult to know how funding occurs in these [29, 32], and other volunteer programs but they are likely to be grant based or from donations thereby making sustainability an ongoing issue [12]. The development of many BD models has been to address inequity of access to healthcare and as such, may be volunteer based [20, 29, 32]. DDs have also followed this path, with some trying to accommodate the disadvantaged by having a sliding scale fee [3]. Two studies describe DD volunteer programs and while both integrate with specialist palliative care, they are quite different models [25, 33]. The Baylor Doula Program is a model of providing support for those with limited means, screened and trained by a palliative care team and then matched with clients [33], whereas the palliative care doula is an expert or advanced practice nurse, who in a volunteer role provides ministry for those who are dying [25].

Once again, role specialisation aligned with BDs has evolved over the years with examples including abortion doulas [34], BDs working with women with an intellectual disability [35], BDs working with incarcerated women [36], and BDs working with pregnant adolescents [37]. Two examples of specialised BD integration into medical care include abortion doulas and support of patients admitted to intensive care units. In each case the BD has provided psychosocial care and support while the HCP focussed on the clinical work [38, 39]. Many DDs have already informally established themselves in specialised roles by working in a way that is comfortable to them, for example only working in bereavement care [3].

### Implications

There is complexity associated with the lack of DD registration, standardisation of DD training programs and working practices [3]. It is unsurprising then, that this lack of consensus in formalising any aspects of the DD role is also found in this current study examining the views of the DD training organisations themselves. Similarities are drawn to the BD role, with Young [40], calling this *navigating the uncertain terrain of an emerging occupation* (p. 306) noting the same ambivalence in views, with tensions apparent between the individuality of care work and the need for professionalisation (or rather legitimisation) of the role. Interestingly though, BD models have evolved and grown without any formal registration or education requirements.

It is likely that in the future the DD role will be formally recognised under a national registration scheme and DD training will be required via a certificate course with steps in place in Australia, at least, for this to happen [12]. Implications lie therein for DD training organisations and DDs themselves, although not all DD training organisations will take this step, with contention regarding the need for such standardisation, and an overriding need to provide flexible and responsive services.

Consideration regarding DDs hired as employees or contractors by a health organisation will likely require some formal processes in place in order for this to happen. This may also be required for incorporation of the DD role into private health insurance options or reimbursement via Medicare. Formal models that employ DDs such as in hospital or in the community will potentially also require funding streams with demonstrated cost benefits shown in BD models [41]. Another consideration is that DDs can currently be engaged via the Australian National Disability Insurance Scheme, a model that could arguably also be considered for community aged care packages. Gift packages can also be purchased for DD services in Australia, a consideration perhaps for consumers. Yet, it will take more than a groundswell approach from DDs themselves to enable these to take place.

The consumer voice also ought to be considered, as no previous studies have been conducted that include the views of consumers who have engaged a DD. Evaluation of the BD role by women who have employed a BD have shown positive effects such as satisfaction and empowerment, although this was in the face of a perceived inflexible health care system [42]. Poor attitudes of midwives are also reported towards BDs at the birth which in turn negatively impacts the mother [43]. When looking to end-of-life care it is also evident that consumer experiences are important to inform policy or to design and /or refine service models [44]. Understanding the experiences of DD consumers is an important future research direction.

### Strengths and limitations

This study is the first to elicit the collective views of DD training organisations internationally regarding DD models of care and working practices. Nonetheless, there are limitations in this study that require consideration. Differences inherent in the health and social care systems of each country could contribute to differing views on the integration or DD role enactment and is also recognised as a limitation of this study. However, these differences could equally be the individual opinions of DD training organisation representatives. Another limitation is that the specific role of the survey respondent in the DD organisation is not explicitly known. Owners, managers, and trainers in a DD training organisation may have varied opinions on the questions asked. It is also unknown how DD training organisations who did not respond or participate would differ from those who did. Despite significant efforts in trying to identify and contact DD training organisations only a small number provided responses. Snowball sampling may have affected the range and type of responses received as it may have reflected collective groupings of training organisations. The five countries represented in the responses are “Western industrial nations” which may also influence the nature of the training approaches and issues. Non-Western non-industrial nations may not offer DD training in the same way, if at all. Also to consider is the vast differences in the cited numbers of DDs trained in each country, and whether this includes birth doulas. If not, then the implications of this rapid increase in the numbers of DDs training could be further explored.

## Conclusion

Death doula training organisations, by default, shape the DD role in how it is enacted and managed, and how the role works and interacts within health and social care systems. This study captures the views of these organisations looking at potential ways forward for DD models of care. Findings indicate that many DD training organisations only see the role as independent, allowing what they see as flexibility in how the role plays out for clients and families. Others are agreeable to DDs integrating into models of care that can support, promote and progress the role, but with caveats around the loss of role independence and being subjected to rules, regulations and oversight. It is likely that a DD model of care will see the DD as contractor rather than integrated into health and social care systems. If there are any discussions regarding the DD role going forward, such as via policy and planning, then the consumer voice needs to be included, especially those who have employed a DD to help care for someone who has died.

## Data Availability

The datasets used and/or analysed during the current study are available from the corresponding author on reasonable request.

## Abbreviations

AUS: Australia
BD: Birth Doula
CAN: Canada
DD: Death Doula
HCP: Health Care Professional
NZ: New Zealand
SWED: Sweden
UK: The United Kingdom
US: The United States
